# The Dammed and the Saved: a Conservation Triage Framework for Wetlands under Climate Change in the Murray–Darling Basin, Australia

**DOI:** 10.1007/s00267-022-01692-x

**Published:** 2022-08-13

**Authors:** Vivienne Schweizer, Matthew J. Colloff, Jamie Pittock

**Affiliations:** Fenner School of Environment and Society, Australian National University, Canberra, ACT 2601 Australia

**Keywords:** Environmental flows, Water reform policy, Wetland conservation, Environmental trade-offs, Decision context, Values, rules and knowledge

## Abstract

As the impacts of climate change and water demands from irrigation continue to increase in the Murray–Darling Basin, water for the environment is becoming more scarce and the ecological conditions of many wetlands is poor. With water scarcity, conservation triage is becoming an increasingly relevant management option for environmental watering of wetlands. However, triage is controversial; being considered contrary to current conservation objectives and practices. We assessed environmental watering at two Ramsar wetlands, Macquarie Marshes and Gunbower Forest, based on international environmental treaty obligations and domestic policy settings, changes to flow regimes, wetland condition and current management. Triage decision making was found to be in tacit use at Macquarie Marshes, based on ‘rules of thumb’ and experiential ecohydrological knowledge, whereas formal environmental watering planning formed the basis for triage decision making at Gunbower Forest. We developed a framework for conservation triage of wetlands in the Murray–Darling Basin to stimulate change in the decision context for wetland conservation and adaptation under climate change. Conservation triage entails reframing of relationships between people and nature and values, rules and knowledge used by stakeholders. Because water is the medium by which wetland conservation outcomes eventuate, trade-offs between competing water uses can be realised with the triage framework.

## Introduction

Human population pressure, economic development and expansion of irrigated agriculture have driven increased global demand for water. Accordingly, increasing water scarcity, declining water quality and altered flow regimes have occurred in many river basins and pressures on water resources have synergised with the negative effects of climate change (Vörösmarty et al. 2010; Greve et al. 2018). Increasing consumptive water demand means less water, and of poorer quality, for the environment (van Vliet et al. 2021). Trans-boundary river basins exemplify the problem of diverse stakeholder groups with competing interests, where high diversions have led to greater water scarcity for consumptive use, altered flow and flood regimes and poor ecological condition of wetlands and their biota (Vörösmarty et al. 2010; Grafton et al. 2013). Herein, we use the Ramsar Convention on Wetlands definition of wetlands, which includes rivers (UNESCO 1994). Declines in ecological condition of wetlands have occurred primarily due to excessive water diversions, combined with the effects of climate change(Pittock and Finlayson 2011; Grafton et al. 2013), resulting in altered flow regimes. As the conservation of wetlands has become increasingly challenging and contested (Green et al. 2017), so priority actions are being considered that go beyond just formal designation in protected areas, which has been considered ‘inconsistently effective’ (Tickner et al. 2020, p. 334). However, the condition of sites listed under the Ramsar Convention was significantly better than of wetlands generally (Davidson et al. 2019).

The flow regime is the major determinant of the structure and function of wetland ecosystems. Every wetland has its characteristic natural flow regime (Poff et al. 2010) and each flow-dependent species has specific environmental water requirements as a consequence of their adaptations to the flow regime (Roberts and Marston 2011; Rogers and Ralph 2011). Reductions or increases in the frequency, extent, magnitude and duration of different types of flow events (cease-to-flow, low-, freshing-, high- and overbank flows) and changes in their seasonal occurrence, can result in the water requirements of certain wetland biota no longer being met. Population recruitment and maintenance then decline and species eventually become locally extinct, replaced by those whose water requirements better match the new flow regime, including invasive, introduced species (Bunn and Arthington 2002). Arid and semi-arid wetlands alternate between dry and wet phases of varying frequency, regularity and duration and ecosystem resilience is dependent on the capacity of the wetland biota to survive prolonged dry phases, grow and reproduce during wet phases and then return to dry phase survival strategies following flood recession (Colloff and Baldwin 2010). If dry periods become more frequent and prolonged, wetlands, or at least those parts of wetland complexes where changes in flow regimes are most pronounced, may transition to dryland ecosystems, commencing with declines in cover of flow-dependent vegetation and increased extent of terrestrial species and bare ground (Mason et al. 2022).

In regulated river basins, the ecological condition of wetlands is managed primarily by the supply of environmental flows (Arthington 2012; Acreman et al. 2014). Under climate change, higher rates of evaporation due to drying, warming conditions, even with the same or greater rainfall, mean that larger volumes of environmental flows are likely to be required to deliver equivalent ecological benefits, while higher evapotranspiration from irrigated crops drives greater demand for water diversions, thus increasing the gap between demand and supply and contributing to the contestation between water for irrigation and the environment.

The Murray–Darling Basin (hereafter ‘the Basin’) in south-eastern Australia is one such regulated river basin in which water for the environment has become increasingly scarce and contested due to demands from irrigation (Colloff and Pittock 2022) and effects of climate change (MDBA 2020). The Basin is characterised by its inland draining nature, with high discharge losses over lengthy arid floodplain traverses of the major rivers, many terminating in extensive wetlands supporting high biodiversity, including 200 wetlands of national importance (DAWE 2020) and 16 Ramsar sites, of which 12 are managed with environmental flows (Kirsch et al. 2021; Chen et al. 2021). Projections of the effects of climate change in the Basin include lower rainfall, especially in the southern Basin, decreased soil moisture and runoff and longer, more frequent and severe droughts interspersed by more intense rainfall events and flooding (Whetton and Chiew 2021).

The Basin has been the focus of a long process of water reform (Beresford 2021). The rapid expansion of irrigation during the 1950s to the mid-1980s led to a four-fold increase in irrigation diversions (Colloff et al. 2015, Fig. 1 therein) and recognition in the 1990s that water resources were over-allocated, accounting for about half the available surface water (CSIRO 2008a, p. 32). This situation stimulated increased inter-jurisdictional collaboration, the Council of Australian Governments (CoAG) reforms in 1994 (Marshall and Alexandra 2016), a Basin-wide cap on diversions in 1995 and the development of the National Water Initiative (CoAG 2004), which forms the basis of current water reform policy. These changes set the scene for the *Water Act* (Commonwealth of Australia 2007) and the Murray–Darling Basin Plan (Commonwealth of Australia 2012), which form the legislative basis for current water reforms.

**Fig. 1:**
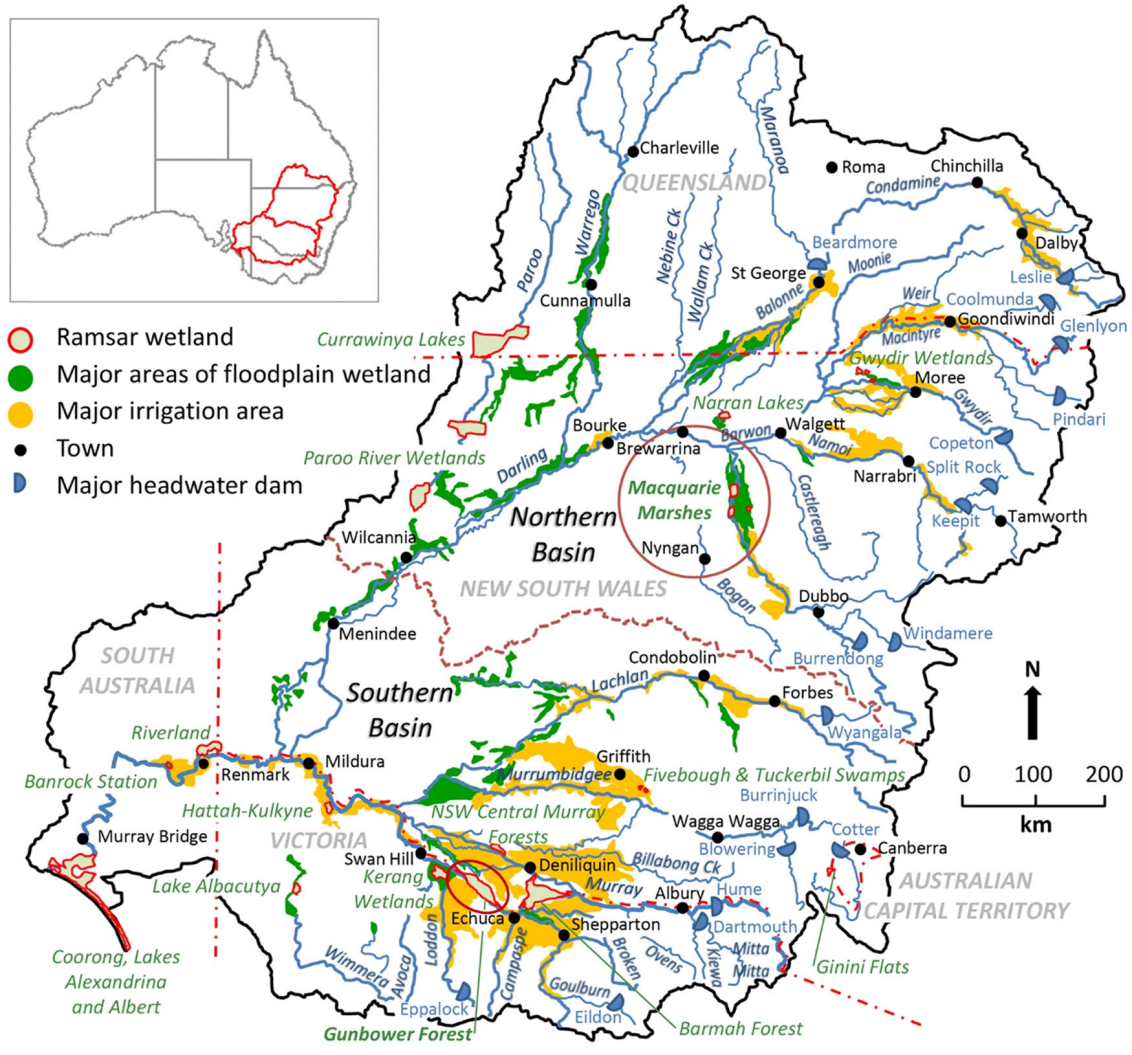
Map of the Murray–Darling Basin showing major wetlands, irrigation districts and locations of Macquarie Marshes and Gunbower Forest (circled)

The Murray–Darling Basin Plan (hereafter ‘the Basin Plan’) guides a $AU 13 billion program to return water from irrigation to the environment. The Basin Plan is a legislative instrument of the *Water Act*, which gains its constitutional legitimacy largely through international environmental treaties: the Ramsar Convention on Wetlands (UNESCO 1994), the Convention on Biological Diversity (SCBD 1992), the Convention on the Conservation of Migratory Species of Wild Animals (the Bonn Convention; CMSS 2020) and bilateral agreements on migratory birds with Japan (JAMBA; DFAT 1981), China (CAMBA; DFAT 1988) and South Korea (ROKAMBA; DFAT 2007). These treaties contain explicit provisions for the conservation of wetlands and biodiversity. Accordingly, the Commonwealth government’s obligations include the provision of water for the environment, the maintenance of the ecological character of wetlands and the conservation of biological diversity and are undertaken via the *Water Act*, the Basin Plan and the *Environment Protection and Biodiversity Conservation Act* (EPBC Act; Commonwealth of Australia 1999). Central to the Basin Plan is the implementation of a Sustainable Diversion Limit (SDL) on the volume of water that can be taken from rivers in each catchment for consumptive use, underpinned by an Environmentally Sustainable Level of Take (ESLT), which came into effect in 2019. In 2009, the volume of water required to maintain wetlands and rivers was estimated at 3000–7600 GL per year (MDBA 2010, p. 110). Since then, this volume has been steadily stepped down to 2075 GL per year, and the Basin-wide SDL increased accordingly, following repeated policy interventions due to droughts, concerns over water security and political contestation over impacts of less water on irrigation communities (Colloff and Pittock 2022). The average volume of held environmental water released (2012–2013 to 2018–2019) was estimated at 1905 GL y^−1^ (Chen et al. 2021), updated to 1897 GL y^−1^ with 2019–2020 and 2020–2021 included (Colloff and Pittock 2022); considerably less than the likely environmental water requirements of the wetlands in the Basin (Prosser et al. 2012, pp. 20–94).

As water for the environment becomes more scarce and contested, so environmental water managers are forced to make trade-offs about how, when and where to supply environmental flows. Many Basin wetlands are in poor condition (Davies et al. 2010) and their flow regimes have been greatly modified from natural conditions (Sims et al. 2012). Decisions on environmental watering are becoming more complex and managers are already exercising ‘triage by default’, whereby environmental watering is prioritised mostly to in-channel flows, smaller wetlands and a fraction of the lower-lying parts of large wetlands that can be flooded with the limited volume of water available to maintain their condition, but other wetland sites tend to miss out (Chen et al. 2021, p. 615). Decisions have to be made based on operational limits of water availability and what ecological benefits can realistically be achieved. The median decline in runoff by mid-century caused by climate change is projected to be similar to the volume of water returned to the environment under the Basin Plan (Whetton and Chiew 2021). Under conditions of increasing water scarcity, we consider it is unrealistic to assume that substantially more water can be allocated to the environment to meet the needs of wetlands in the Basin.

Conservation triage is the process whereby resources are inadequate to achieve all desired objectives and are allocated to those populations, species or ecosystems considered to have a high chance of persistence or deemed of high conservation value. Like medical triage, conservation triage involves more than just prioritisation based on the likely success of interventions (Kennedy et al. 1996). Resources are made available only to those entities deemed most in need by withholding them from those likely to decline, even if resources were provided, and deferring them from those where damage is slight. This process requires the sorting (French, *trier*; to sort) of entities, according to standardised, transparent and repeatable decision-making criteria (Bottrill et al. 2008; Dallimer and Stringer 2018), to achieve the greatest good for the largest number of entities where resources are limited. However, available resources may improve or decline, thus requiring the re-assessment of entities into classes for which resources are provided, withheld or deferred. Furthermore, the level of threat and the probability of ecosystem persistence and recovery adds to the complex of decision making scenarios, ranging from the requirement for immediate management interventions for protection and restoration through to prevention and mitigation of the effects of threatening processes (Hobbs and Kristjanson 2003). Conservation triage is distinctive in the recognition that some entities contribute more than others to particular objectives (e.g. ecosystem functions and services, maintenance of habitat or meta-population viability). Medical triage does not involve sorting by the contribution of individuals.

Critics claim conservation triage signals it is socially acceptable to consider some entities as expendable (Buckley 2016). Yet species extinctions and declining ecological condition will occur under existing management that has not adapted to address the negative impacts of climate change. Under such conditions, striving for recovery of all species and addressing the root causes of extinction and environmental decline (Wiedenfeld et al. 2021) is unrealistic, regardless of whether species are considered to have a fundamental right to exist. Opponents of conservation do not need to invoke triage to argue that environmental degradation is an acceptable price to pay for development.

Triage is likely to become increasingly relevant in wetland conservation. Because water is the medium by which most conservation outcomes are achieved, opportunity costs arise and trade-off decisions need to be made between competing water uses. The current decision context for trade-offs on environmental flows to wetlands focuses on the how, when and where, but does not address the ‘why’ in a systematic way. A triage framework places the ‘why’ as central to changing the decision context, together with the systems of values, rules and knowledge that are deemed credible, legitimate and important under climate change (Gorddard et al. 2016; Colloff et al. 2018).

In this paper we address the shortfall of environmental water availability by considering options for conservation triage of wetlands. We first assessed key elements of policy and practice in environmental water management in the Basin, including changes to flow regimes, obligations under international environmental treaties and domestic policy settings, wetland condition, water availability under a warming climate and increasing demand for irrigation diversions in relation to two case study wetlands. We then applied our findings from the case studies on these elements to develop a decision framework as a basis for determining whether a site should be subject to environmental watering triage.

## Methods

### Background – Environmental Watering under the Basin Plan

An objective of the Basin Plan is to protect and restore ‘water-dependent ecosystems’ and their ecosystem functions, ensure they are resilient to climate change and other threats and co-ordinate management of environmental watering (Commonwealth of Australia 2012, S5.03). The reduction in SDLs for each catchment is via Water Resource Plans (WRPs) to be implemented by the Basin States (Queensland, New South Wales, Victoria, South Australia) and the Australian Capital Territory.

The Commonwealth Environmental Water Office (CEWO) manages environmental water recovered from irrigators in collaboration with the States and Territory. Environmental objectives include maintaining river connectivity, native vegetation and populations of waterbirds and fishes, as well as conserving ecologically important wetlands (MDBA, 2019). Environmental water to achieve these objectives includes water held by CEWO, New South Wales, Victoria and South Australia and jointly with MDBA under The Living Murray Program (MDBA 2011). In Queensland, environmental water is protected in WRPs for each catchment via rules on access to water. The CEWO partners with States and other water holders in sharing contributions to environmental water for release in most river valleys (cf. CEWO 2013, pp. 8–10, 19–21 for operational details). Annual environmental watering priorities are set by the MDBA, intended to support the Basin-wide Environmental Watering Strategy (EWS; MDBA 2019) and the ecological objectives in the Basin Plan.

Operational constraints on environmental watering include the volume available, based on antecedent wet or dry years, the long-term average annual yield of Commonwealth water entitlements (Chen et al. 2021) and how much water can be released without flooding private land (Kahan et al. 2021). Impacts of climate change are already apparent, with extended droughts and reduced winter rainfall leading to declining in inflows over the last 20 years; 39 percent below the long-term average (MDBA 2020). The Basin was in drought for 70% of the time (14 years) between 2000–2020, compared with 35% between 1895–2000 (Colloff and Pittock 2022).

### Case Studies and an Environmental Watering Triage Framework

We used Macquarie Marshes in the northern Basin and Gunbower Forest in the south as case studies (Fig. 1). Our assessment was based on peer-reviewed scientific literature, technical reports, policy documents, management plans, media reports and unpublished databases. We also developed and extended our consultation and collaboration with environmental water managers at Macquarie Marshes and Gunbower Forest, as well as staff from CEWO, MDBA and the New South Wales Department of Planning and Environment (DPE).

As a basis for the triage framework, we examined five elements of wetland policy and management: (1) international environmental treaty obligations, including the Ramsar Convention, the Convention on Biological Diversity and agreements on migratory species; (2) national conservation legislation: the *EPBC Act*, the *Water Act* and the Basin Plan regarding the object ‘to protect and restore the ecosystems, natural habitats and species that are reliant on the Basin water resources’ (Commonwealth of Australia 2007, S21(2)(b)); (3) changes to historical flow regimes caused by river regulation, irrigation diversions and climate change; (4) wetland ecological condition and (5) recent environmental watering actions. By assessing these elements we could order them in a decision framework as a basis for determining whether a wetland should be subject to triage. We then sought feedback and revised the framework accordingly. Consultation with CEWO and MDBA staff included workshop discussions (April, August and November, 2021).

**Fig. 2:**
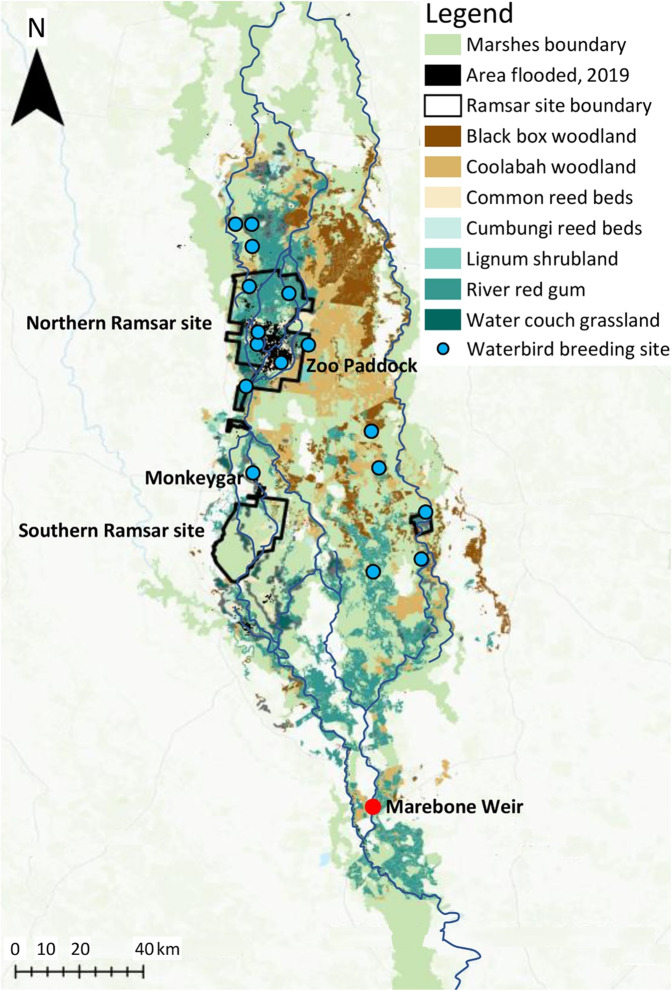
Map of the Macquarie Marshes showing vegetation classes (Bowen et al. 2019; DPIE 2014; DPIE 2020), extent of flooding in 2019 and waterbird breeding sites (OEH 2012, Fig. 8 therein, based on Kingsford and Auld 2005)

Macquarie Marshes and Gunbower Forest contain extensive stands of River Red Gum (*Eucalyptus camaldulensis*) floodplain forest and woodland (Figs. 2 and 4), are listed as wetlands of international importance under the Ramsar Convention and receive environmental flows managed by CEWO and State government agencies. However, their ecological character, ecohydrology and water management are sufficiently different to identify and test a range of elements in the triage framework. Below, we outline basic biophysical characteristics of each site. More detail is given in the sections on the element of wetland policy and management.

**Fig. 3:**
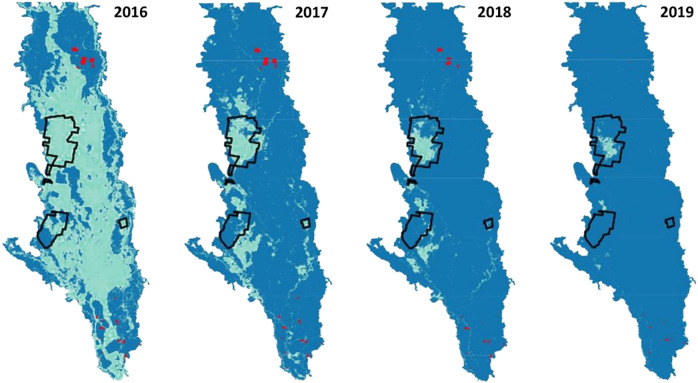
Maps of the extent of flooding at Macquarie Marshes, 2016–2019. Blue = extent of marshes; green = areas flooded; black outline = boundaries of Ramsar site; red = on-farm storages containing water (DPIE 2020)

Macquarie Marshes is an extensive, deltaic wetland in the northern Basin in New South Wales (Figs. 1 and 2), occupying ca. 2000 km^2^ of low-gradient, semi-arid lowland floodplain and formed by anastomosing channels of low mean annual discharge but highly variable flow (Ralph and Hesse 2010). Channels are unstable, being prone to avulsion: the geomorphic relocation of sediments and flows (Ralph et al. 2016). At the southern, upstream end of the Marshes is an extensive area of irrigated cotton production where irrigation diversions and floodplain water harvesting (diversion and storage of overland flows in on-farm dams) has reduced inflows to the Macquarie River (Brown et al. 2022).

**Fig. 4:**
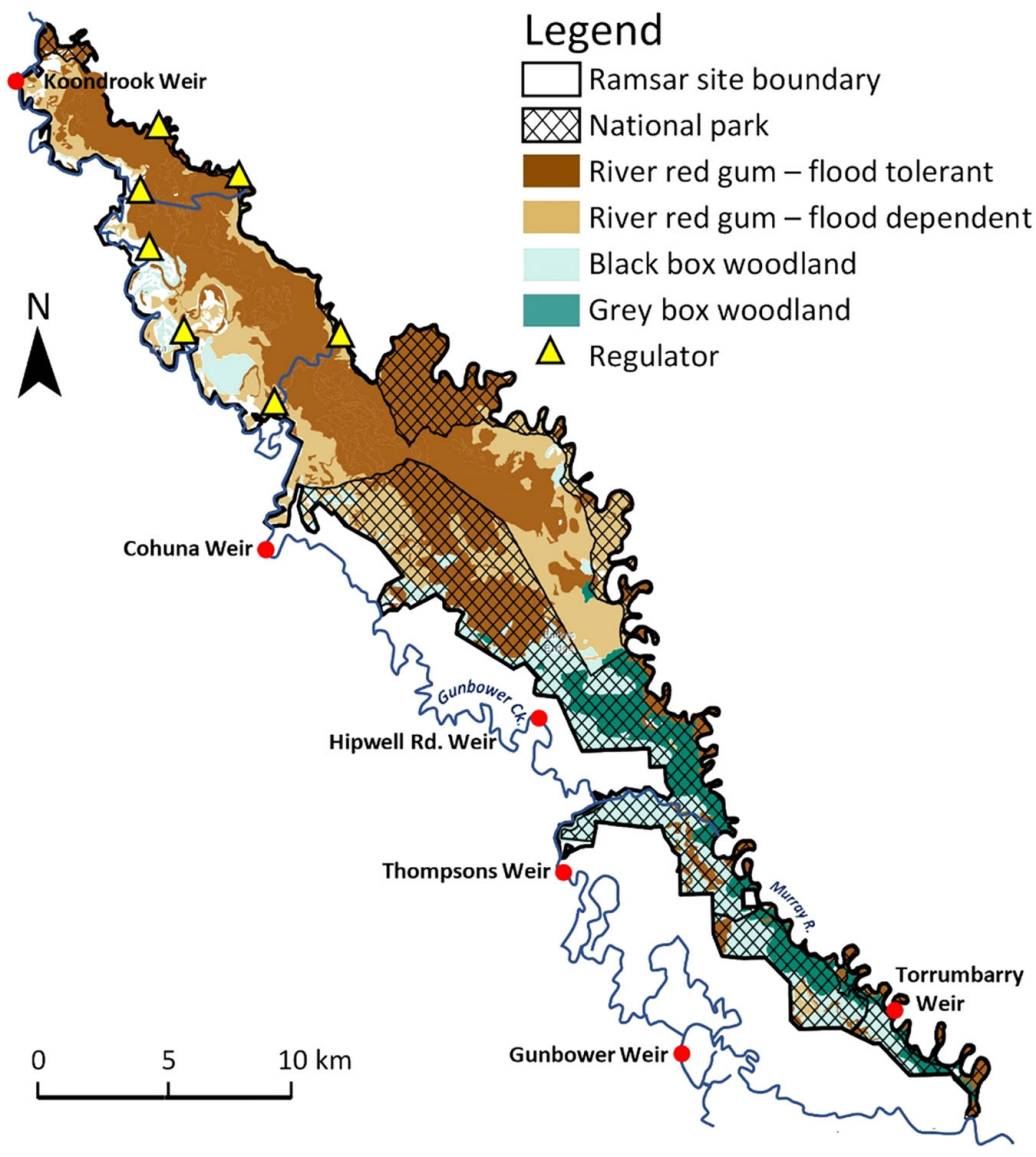
Map of Gunbower Forest showing vegetation classes (North Central Catchment Management Authority [NCCMA], unpublished data) and environmental infrastructure (NCCMA 2015, Fig. 2 therein)

Gunbower Forest in Victoria is on an island, with the Gunbower Creek anabranch to the south and the River Murray to the north (Figs. 1 and 4). It occupies about 200 km^2^ of lowland floodplain with well-defined braided channels and is part of the Gunbower–Koondrook–Perricoota Forest (ca. 510 km^2^). Flood frequency and duration have declined markedly since river regulation (Cooling et al. 2002; Cooling and SKM 2012). A flood enhancement engineering works program, completed in 2013 but not yet operational, is predicted to allow flooding of 4.7 km^2^ of wetland with less water than was previously required for this inundation extent (MDBA 2012; NCCMA 2014). Gunbower Forest is bordered by irrigated agricultural land, but without extensive floodplain water harvesting.

## Results and Discussion

### International Environmental Treaty Obligations

#### Ramsar convention

Macquarie Marshes Ramsar site was listed in 1986 and covers 19,850 ha or 10% of the Marshes. Under Australian law, a broader area may need to be sympathetically managed to conserve the values of a Ramsar site. The site meets six criteria for Ramsar listing, including being a rare wetland type (intermittent freshwater marsh), supporting endangered species and providing breeding habitat for large populations of waterbirds (Supplementary Table S1; OEH 2012). A notification of change in ecological character was submitted to the Ramsar Secretariat in July 2009, based on declining vegetation condition and extent and a likely change from a semi-permanent to an ephemeral wetland (DEWHA 2010).

Gunbower Forest Ramsar site was listed in 1982 based on four criteria (Supplementary Table S2). At the time, the site was a state forest, so forestry practices have shaped the vegetation communities (Hale and Butcher 2011). Forestry ceased over nearly half the area in 2010 when part of Gunbower Island was designated a national park. The decline in frequency of medium-sized floods that watered most of the River Red Gum forest signals a likely change in ecological character at Gunbower–Koondrook–Perricoota Forest (NRC 2009, p. 190).

#### Convention on Biological Diversity (CBD)

The Macquarie Marshes and Gunbower contain extensive River Red Gum forest and River Red Gum and Black Box *Eucalyptus largiflorens* woodland. We estimated 52 and 42 percent, respectively, of these wetland types are contained within protected areas in the Basin (Table 1), exceeding the CBD target of at least 30 per cent of areas of particular importance for biodiversity conserved within protected areas (SCBD, 2021).

**Table 1:** The extent of wetland classes in the Murray–Darling Basin in relation to their proportion contained within protected areas (national parks, nature reserves, conservation areas, Ramsar sites, and other reserves)

Main wetland type	Total area (ha)	Within protected areas (ha)	Percentage protected
Arid northern & western floodplain wetland	4,672,035	472,771	10.1
River Red Gum & Black Box woodland	732,311	306,209	41.8
Sub-alpine bog	350	350	100
Aquatic grassland & rushland swamp	136,853	10,245	7.5
River Red Gum forest	270,199	140,224	51.9
Estuarine and lower lakes complex	142,530	49,776	34.9
Total	5,954,278	979,575	16.5

In the absence of a standardised classification of wetland types for the Basin, we used the major vegetation formations and classes of Keith (2004) for New South Wales, together with the descriptions of the dominant vegetation community for each of 66 major Basin wetlands, as a basis to derive the main wetland types listed below

#### Agreements on migratory species

The Marshes provide habitat for 17 species listed under the agreements on migratory birds (OEH 2012, p. 14), relevant to the Australian government’s obligations under the Bonn Convention and migratory birds treaties, while Gunbower has seven species (Hale and Butcher 2011, Appendix B therein; Australian Painted Snipe *Rostratula australis* was incorrectly listed). Cattle egret *Ardea ibis*, Eastern Great Egret *A. modesta* and Glossy Ibis *Plegadis falcinellus* are the colonial nesting waterbirds covered by the agreements. Large ibis colonies at Monkeygar and Zoo Paddock are outside the Macquarie Marshes Ramsar site but nearby, as are several other waterbird breeding sites (Fig. 2). Successful breeding of ibis and egrets occurred in 2016–2017 after major floods (Brandis 2017; Fig. 3). A flow threshold of 1500 ML d^−1^ for 30–50 days (Oxley gauge) correlates with a high probability of waterbird breeding, or 110–150 GL discharge (July-December) for breeding initiation (Arthur et al. 2012). The 19 breeding events between 1978 and 2016 (Brandis 2017, p. 9), and one in 2019, indicate thresholds can still be met. At Gunbower Forest, breeding events have been sparse: none between 2007 and 2012 (Kingsford et al. 2013, p. 144) and two between 2007 and 2017 (MDBA 2018, p. 18).

### National Conservation Legislation

The Macquarie Marshes supports two species of fishes (Murray Cod *Maccullochella peelii peelii* and Silver Perch *Bidyanus bidyanus*) and two species of waterbirds (Australasian Bittern *Botaurus poiciloptilus* and Australian Painted Snipe) listed as threatened under the *EPBC Act* (Supplementary Table S3). The fishes are the only two threatened species at Gunbower Forest. Distribution records of the four threatened species at the Marshes (Supplementary Fig. S1) indicate the entire area represents critical habitat for them. At Gunbower, there are records of the fishes along the north-east reach of the River Murray (Supplementary Fig. S2) which is deemed critical habitat.

### Historical Changes to Flow Regimes

Macquarie Marshes has been transformed by livestock grazing, irrigation diversions, land clearance and river regulation, with eight weirs and nine upstream dams (Kingsford 2000; OEH 2012). Before river regulation and diversions in the 1960s, half the flow at Dubbo (100 km upstream) reached the Marshes, falling to one fifth by 1995 and wetland extent has halved (Kingsford and Thomas 1995). The Marshes ranked 19/40 Basin wetlands for divergence of flows from natural conditions (Sims et al. 2012). End-of-system flows declined from 777 GL y^−1^ under historical climate and no development to 583 GL y^−1^ under recent climate and current development (CSIRO 2008a). It is unlikely the ecological character can be maintained in a warming climate with current flows and environmental watering (Capon et al. 2018).

Flood frequency and duration at Gunbower Forest has declined markedly due to river regulation and irrigation diversions, causing loss of flood-dependent vegetation and encroachment of less flood-dependent species (Cooling et al. 2002). Since river regulation, frequency of flooding flows (>36–45 GL d^−1^ at Torrumbarry Weir) have declined from every 1.5 years to 4 years and large floods from every 2.4 years to 12.5 years (Cooling and SKM 2012, p. 16). Flood volumes under current climate and water resources development are 17% of those under historical climate and no water resource development (CSIRO 2008b, p. 168).

### Wetland Condition

At Macquarie Marshes, reductions in flood extent and frequency since 1978 have caused declines in extent and condition of wetland vegetation (Thomas et al. 2010; Bowen 2019; Bowen et al. 2019). Probability of flooding in the previous five years had strongest explanatory power for River Red Gum condition, but less flooding has caused extensive dieback (Catelotti et al. 2015). River Red Gum forest requires floods at least every three years for maintenance (Roberts and Marsden 2011, p. 49) but can survive extended dry periods (Doody et al. 2015). Floods during 2020–2022 helped maintain condition within the Ramsar site (Fig. 3). Common Reed *Phragmites australis* beds at the northern Ramsar site were flooded annually between 2016 and 2019, meeting their water requirements (flooding every one to two years; Fig. 3). However, reed beds in the southern Ramsar site, which have declined in extent (OEH 2012, p. 82), were flooded only once between 2016 and 2019 and were in poor condition prior to drought-breaking floods in 2020.

**Fig. 5:**
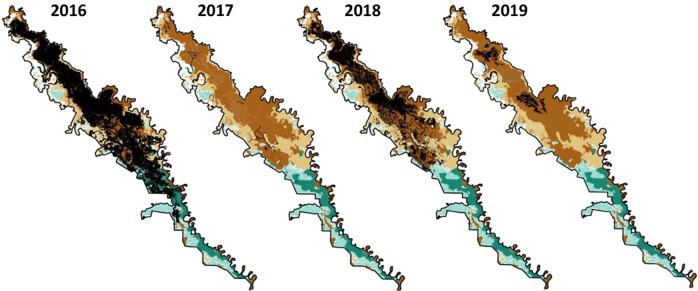
Maps of the extent of flooding (in black) at Gunbower Forest, 2016–2019 (North Central Catchment Management Authority, unpublished data)

Three quarters of the extent of Gunbower is River Red Gum forest (Cooling et al. 2002), with either flood-dependent understory, requiring floods every one to two years or ‘flood-tolerant’ (i.e. less dependent on flooding) requiring floods every three or four years (Fig. 4; Hale and Butcher 2011). During 2016–19 these requirements were met, with near-complete inundation of the flood-dependent community in 2016 and 2018 (Fig. 5). Black Box woodland in the west and south (Fig. 4) requires flooding every three to seven years for growth and flowering (Roberts and Marston 2011, p. 15). Almost half was flooded in 2016 (Fig. 5). The frequency and extent of floods might indicate the Gunbower Forest Ramsar site is in good condition. However, vegetation of nearby Gunbower Creek is considered in moderate to poor condition and declining, with tree deaths, bank denudation and extensive aquatic weeds (NCCMA 2015, pp. 64–65). Tree deaths along the creek may be due to excess water as a consequence of artificially high flows for water supply purposes (Mall et al. 2022).

### Environmental Watering

Between 2009–10 and 2018–19 the Macquarie catchment received an average of 114 GL y^−1^ of environmental water (Colloff and Pittock 2022, Table S1 therein), but during the 2017–2020 drought the northern Ramsar site was flooded annually while the southern site remained dry (Fig. 3). Only 4.6 GL of supplementary water was delivered to the Macquarie River and Marshes in 2019–20, compared with 80 GL in 2016–17 (CEWO 2021a); a wet year during which most of the Marshes flooded (Fig. 3). The lower Macquarie River ceased to flow for 14 months prior to drought-breaking rains of February-April 2020 (CEWO 2021b), after which much of the Marshes flooded. To continue drought recovery, 137 GL was delivered in 2020–21, flooding 21,600 ha, including water couch and reedbeds in the northern Ramsar site (CEWO 2021c).

Extensive flooding occurred in 2016 at Gunbower Forest (ca. 10,000 ha; Fig. 5) due to high rainfall (August-October) and 28 GL of environmental water was delivered to Gunbower Creek (CEWO 2021d). Almost no flooding occurred in the drought year of 2017–18 and sedges and reedbeds were in poor condition (VEWH 2019, p. 54). Flooding of a third of the forest in 2018–19 (Fig. 5) followed delivery of 62 GL (VEWH 2019, p. 83), plus 79 GL of Commonwealth water released from Hume Dam to support Murray floodplains. In 2019–20, 23 GL was delivered to Gunbower Creek (VEWH 2020, p. 94).

### Summary and Synthesis

#### Macquarie Marshes

Environmental watering has been prioritised for the northern Ramsar site, particularly for reedbeds that require watering every one or two years. The southern Ramsar site has effectively been subject to triage, having received no environmental water from 2017 to 2020. Drying intensified after 2016–17 and the cover of wetland plant species declined and the extent of bare ground increased (Mason et al. 2022). Triage options need to be assessed against current and future water availability, and what can be achieved. Meeting at least one Ramsar criterion, such as provision of waterbird habitat, is one option, since this has been a consistent objective (DECCW 2010). Yet several breeding sites are outside Ramsar boundaries (Fig. 2; Kingsford and Auld 2005). The physical geography of the Marshes, with channels subject to avulsion and formation of ‘wandering wetlands’ (Ralph et al. 2016), limits how water can be targeted and questions the adaptability of hard boundaries for protected areas.

Prioritising the watering of vegetation that forms waterbird habitat, irrespective of Ramsar boundaries, is justifiable under triage. This approach accords with how managers make watering decisions. Triage involves a long-term perspective, including time since last watering, flow regimes needed to maintain a mosaic of vegetation communities, the ability to deliver water to sites and whether watering is targeted to a few sites or spread broadly (Tim Hosking, personal communication, September 2021). Maintaining vegetation communities is central to these decisions. Watering only selected communities within a wetland is a form of triage.

Conserving the ecological character of only a portion of the designated Ramsar sites is a clear abrogation of Australia’s commitments under the Ramsar Convention (Pittock et al. 2010). Distinguishing causation for limited watering of these sites due to direct management choices versus impacts of climate change could be debated. It is clear that, to date, at both sites governments have chosen to limit delivery of environmental flows in favour of irrigated agriculture (Colloff and Pittock 2022), which would only be justified under the Ramsar Convention if this choice was declared to be in Australia’s ‘urgent national interest’. Nevertheless, climate change is diminishing river inflows in the Basin (MDBA 2020, p. 21) and triage decisions on which wetland and socio-economic values are sustained have become inevitable. Under climate change, maintaining the ecological character of Ramsar wetlands, as detailed at the time of listing, is perceived as increasingly unrealistic (Pittock et al. 2010; Pritchard 2021; Partridge and Finlayson 2022), as this approach does not include consideration of the spatio-temporal dynamics of wetland complexes. However, the Ramsar Convention makes allowance for changes in ecological character, with restoration as the preferred management option. If restoration of whole or part of a wetland complex is not possible, a site may remain a Ramsar wetland if one or more listing criteria remain fulfilled (Pittock et al. 2010, p. 422). This approach provides at least some flexibility to allow for continued conservation and management under the Ramsar framework. Changes in ecological character of Ramsar wetlands have been managed, in part, by setting thresholds of potential concern and limits of acceptable change as part of ecological character descriptions and adaptive management plans (Biggs and Rogers 2003; Rogers et al. 2013; Roux and Foxcroft 2011). However, addressing climate-related changes in ecological character remain an unresolved issue for the Ramsar Convention, including the definition of baseline conditions, identifying the extent of change that warrants a management response and distinguishing anthropogenic drivers of change from natural ones (Finlayson et al. 2017; Pritchard 2021). Objectives for wetland management can and are being set and implemented under climate change, including environmental triage as part of strategic adaptive management, yet the Ramsar Convention has yet to effectively address adaptation to climate change (Finlayson et al. 2017).

How wetlands respond to climate change depends on ecosystem properties that confer resilience, characterised by the capacity of wetlands to transition to and from wet and dry phases (Colloff and Baldwin 2010). These properties include response diversity of wetland plants: their persistence under environmental extremes (Lavorel et al. 2015). Many wetland plants are resilient to drought (Sandi et al. 2020). River Red Gum tolerates at least nine years without flooding and then recovers following watering (Doody et al. 2015). Emergent macrophytes persist via bulbs, corms, rhizomes, tubers and seedbanks, enabling rapid recolonisation after floods, providing a source of carbon and energy to survive the dry phase and then drive biogeochemical transformations upon return of the next wet phase (Baldwin et al. 2013). The impact of climate change on the Marshes is uncertain. Longer-term changes and natural variations in drought and flood are likely to amplify existing effects on wetland vegetation (Sandi et al. 2020; Saintilan et al. 2021), adding complexity to future water management decisions.

#### Gunbower Forest

Prioritisation of wetlands for environmental watering is formalised in an environmental water management plan, with clear objectives under different scenarios of water availability (MDBA 2012). Under water scarcity, permanent wetlands have highest priority, then semi-permanent wetlands. As more water becomes available, River Red Rum forest with flood-dependent understorey is included and in a wet scenario, River Red Gum with less flood-dependent understorey and small areas of Black Box (MDBA 2012, p. 23). As environmental water becomes scarcer, prioritisation in the plan becomes congruent with a triage approach, enabled by delivery of water to targeted wetlands via the environmental infrastructure.

River Red Gum communities consistently receive their environmental water requirements. The remaining Black Box woodland receives little environmental water. Yet this is an important vegetation community of high biodiversity value, though much of it is in poor condition (Overton et al. 2018). Black Box occurs on the upper floodplain, mostly above the 1-in-10 year flood elevation level, so rarely receives environmental water, even though there are Basin-wide objectives for maintaining its extent (MDBA 2019, Appendix 2 therein). There is little or no regeneration in many Black Box woodlands in the southern Basin (Roberts and Marston 2011, p. 13). The lack of environmental watering constitutes triage by default. Black Box receives environmental watering at Gunbower, but only a ‘minor area’ (not specified) and only under a wet scenario (MDBA 20112, p. 23). Its water requirements will not be met if the frequency of wet scenarios declines further under a drying climate.

Triage is already happening at Gunbower Forest, largely because the environmental infrastructure enables managers to target water to specific wetlands. But other wetlands that cannot be so targeted lose out. The current prioritisation of watering is adequate to ensure that when triage decisions are made they can be justified. However, as the impacts climate change increase, new knowledge on rates and extent of changes in vegetation condition and composition will alter the decision context for environmental watering.

### A Triage Framework for Environmental Watering

**Fig. 6:**
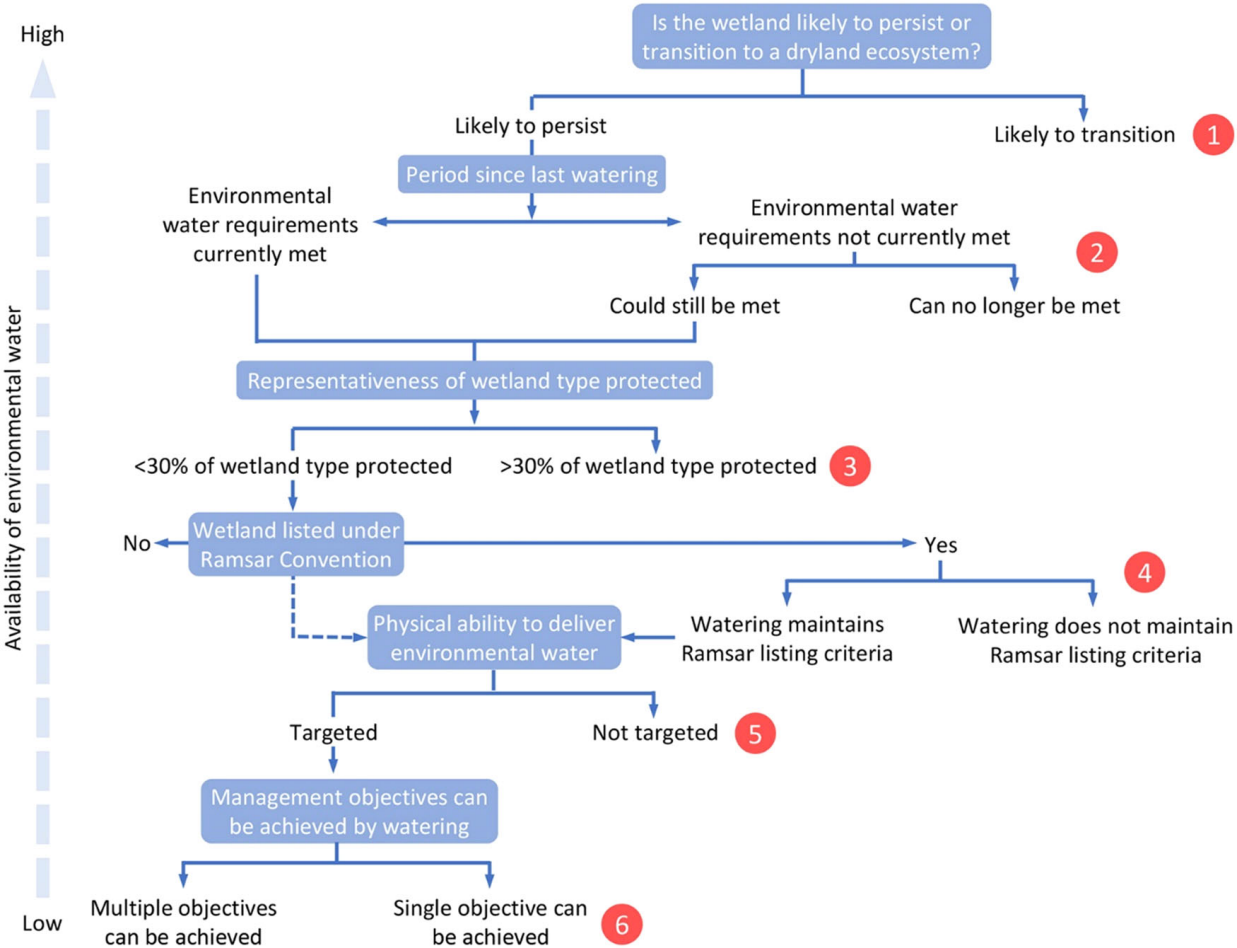
An environmental triage framework for wetlands in the Murray–Darling Basin. Working sequentially, the answer to the questions and their associated focal issues at each decision point determines the level of triage for a particular wetland. The less environmental water available, the higher the triage level (from 1–6) at which decisions need to be made and actioned

Working sequentially, the issues at each decision point in the framework determine the level of triage for a wetland (Fig. 6). The less water available, the lower the level at which decisions are actioned. The first issue is whether the wetland will persist under hotter conditions or transition to a dryland ecosystem. Climate change will cause further shifts in the structure, function and extent of wetlands in the Basin (Saintilan et al. 2013; Colloff et al. 2016). Applying triage at the scale of individual wetlands focuses on what is required to maintain them but also on trade-offs and opportunity costs to other wetlands. If ecological transformation is already occurring, water can be assigned to wetlands more likely to persist. If a wetland is unlikely to persist it should not receive environmental water.

The second issue is whether water requirements of vegetation have been met in the past and, if not, whether they could be met in the future. This assessment helps evaluate ecological condition, including properties that confer ecosystem resilience (Colloff and Baldwin 2010).

The third and fourth issues address international treaties: whether sufficient extent of the wetland type is within protected areas, based on the 30 percent CBD target (SCBD 2021). Under the Ramsar Convention, parties commit to protect all wetlands, although obligations for managing change in ecological character caused by climate change are unclear and need to be revised (Pittock et al. 2010: Finlayson et al. 2017). In theory, Ramsar listing appears to confer no advantage over other wetlands, though in practice Ramsar sites are prioritised because of their ecological significance. We consider they should be prioritised accordingly and because it is impossible to maintain all wetlands with limited environmental water.

The fifth issue involves the practicalities of delivering environmental water: whether it can be targeted to wetland sites of high conservation value or spread broadly, including to sites of low conservation value, incurring seepage and evaporation losses in the process.

The final issue is whether watering is based on meeting one or two management objectives or whether multiple objectives can be met, including cultural and social benefits to local communities and Indigenous Peoples, including their active participation in wetland conservation. Protection of wetlands is central to cultural values and well-being of many Indigenous Peoples in the Basin (Moggridge and Thompson 2022), yet they remain marginalised in decisions on water policy and management (Hartwig et al. 2022).

The triage framework is a basis to build on and adapt. It is not an operational decision system to be implemented directly. We acknowledge that threats from the interaction between climate change and consumptive water use and the uncertainties around ecosystem transition or persistence add to the complexity of decision making under triage scenarios. Addressing these issues requires a broader understanding of the drivers of ecosystem transitions, the range of possible management responses and the functional characteristics of ecosystem resilience of wetlands. Accordingly, we have attempted to make the triage framework specific enough to consider triage decisions for individual wetlands but general enough to be used and adapted for the variety of wetlands in the Basin. The framework is intended to stimulate discussion and change in the decision context for wetland conservation under climate change. This framework could inform deliberations within the Conference of the Contracting Parties of the Ramsar Convention on practical measures for advancing its provisions for wetland conservation under a changing climate.

## Concluding Remarks – Adaptation as Change in the Decision Context

Decision contexts are set by interacting values, rules and knowledge considered credible, legitimate and important by decision makers (Gorddard et al. 2016; Colloff et al. 2018). These interactions determine which options are chosen and how the values, rules and knowledge held by decision makers influence change. Changes to decision contexts on water use involve collaborative exploration of trade-offs and synergies between consumptive and environmental uses, as part of adaptation to climate change (Colloff et al. 2019). Decision systems developed during periods of stasis by a narrow set of rules and values and exclusive scientific and technical knowledge will be maladaptive under conditions of rapid change (Dryzek and Pickering 2019, pp. 27–30).

One way of changing the decision context is by introducing different knowledge, values and rules. Triage decision making at Macquarie Marshes involves ‘unwritten rules’ developed by managers based on their experiential knowledge and supported by their values as conservation practitioners. Their decision making is supported by the Macquarie–Cudgegong Environmental Water Advisory Group (EWAG; DPE 2022). Where resources are contested, the diversity of values, rule and knowledge participants bring to decision making are at least as important as scientific knowledge of the social-ecological system (Leviston et al. 2018). The diversity of the membership of EWAGs ensures a range of values, rules and knowledge are available to inform decisions, including Indigenous perspectives.

Conservation triage inevitably means reduction in the extent of wetlands as they are left to transition to terrestrial, dryland ecosystems. However, these wetlands would transition anyway under business as usual environmental water management, whereby insufficient water to meet their needs would merely slow the rate of transition. Some researchers and environmental managers argue that although altered flow regimes are a major driver of change in wetland structure and function, other drivers are important, as are other management options for wetland conservation. This perspective invokes the concept of ‘complementary measures’, which are management interventions used as adjuncts to environmental flows (Baumgartner et al. 2019). Complementary measures are based on the premise that environmental flows alone are not enough to deliver restoration benefits given the altered state of many wetlands. Complementary measures that can be integrated with environmental watering and include pest control, infrastructure modifications, habitat restoration, addressing cold water pollution, enhancing fish passage, improving water quality, nutrient cycling and sediment transport. However, implementing complementary measures in isolation will be insufficient to achieve large‐scale wetland restoration without integration into environmental flow management and flow recovery programmes (Cresswell et al. 2017).

Because water is the primary medium by which wetland conservation is achieved, there are important trade-offs between competing water uses that do not apply to species-based conservation triage. Such trade-offs can be achieved with the triage framework presented herein in effective, transparent and accountable ways because it is based on common principles for wetland management and is adaptable. For example, water that would have been used to maintain a wetland that is unlikely to persist could instead be used by an irrigation community during a transition phase to adapt to a mixed farming system that uses less irrigation water.

The Basin Plan revision due by 2026 provides an opportunity to include conservation triage into environmental water policy and management. As our understanding improves of how climate change affects wetlands and local communities, such a framework can be adapted and revised. For now, the framework provides a first step in formalising and making transparent the triage decision processes already in use for the conservation of wetlands.

## Supplementary information

Supplementary Information
