# Supplementary material for: The Dammed and the Saved: a Conservation Triage Framework for Wetlands under Climate Change in the Murray–Darling Basin, Australia
Source: Environ Manage. 2022 Aug 13;70(4):549–64. doi: 10.1007/s00267-022-01692-x (PMC9439973; doi:10.1007/s00267-022-01692-x)
Supplement: Supplementary file 1 — Supplementary Information [file 267_2022_1692_MOESM1_ESM.docx]

**Supplementary Material**

Table S1. The criteria met by the Macquarie Marshes for listing under the Ramsar Convention on Wetlands (source: OEH, 2012)

| Criterion | Description | Details |
| --- | --- | --- |
| 1 | Contains a representative, rare, or unique example of a natural or near-natural wetland type found within the appropriate biogeographic region | Macquarie Marshes is one of the largest wetlands in the Basin and has an unique range of wetland vegetation communities, including river red gum forest and woodlands, common reed and cumbungi marshlands and water couch aquatic grasslands. These communities support high wetland species diversity including waterbirds, migratory shorebirds, frogs, fish and reptiles |
| 2 | Supports vulnerable, endangered, or critically endangered species or threatened ecological communities | Supports at least four species listed under the *Environmental Protection and Biodiversity Conservation Act* (Cth. 1999) or as endangered or vulnerable under the *Threatened Species Conservation Act* (NSW 1995). The Marshes support two ecological communities listed as endangered under the latter Act |
| 3 | Supports populations of plants and/or animal species important for maintaining the biological diversity of a particular biogeographical region | Macquarie Marshes support one of the most extensive River Red Gum forests and woodlands in the Basin. It is one of only two sites with extensive common reed and cumbungi and water couch aquatic grasslands and has the most southerly distribution of coolabah woodland |
| 4 | Supports plants and/or animal species at a critical stage in their life cycles, or provides refuge during adverse conditions | The Marshes support large breeding colonies of several species of ibis and egret, as well as magpie goose, rufous night heron and royal spoonbill and are an important drought refuge for a wide range of species |
| 5 | Regularly supports 20,000 or more waterbirds; and | The number of nesting waterbirds in large breeding events has been consistently greater than 20,000 individuals |
| 8 | Is an important source of food for fishes, spawning ground, nursery and/or migration path on which fish stocks, either within the wetland or elsewhere, depend | The Marshes support substantial populations of native fishes, including species that move from the main channel to the floodplain to breed during periods of high flows. Other species, such as Murray Cod and Silver Perch breed within channel |

Table S2. The criteria met by Gunbower Forest for listing under the Ramsar Convention on Wetlands (source: Hale and Butcher, 2011)

| Criterion | Description | Details |
| --- | --- | --- |
| 1 | Contains a representative, rare, or unique example of a natural or near-natural wetland type found within the appropriate biogeographic region | Gunbower Forest is part of the second largest river ed gum forest in the MDB, and the size and intact nature of it makes it one of the best representatives of this wetland type |
| 2 | Supports vulnerable, endangered, or critically endangered species or threatened ecological communities; | Gunbower Forest supports the threatened species Murray Cod *Maccullochella peelii* and Silver Perch *Bidyanus bidyanus* |
| 4 | Supports plants and/or animal species at a critical stage in their life cycles, or provides refuge during adverse conditions | Some 48 waterbird species breed in the forest and the wetlands provide important spawning habitat for fishes |
| 8 | Is an important source of food for fishes, spawning ground, nursery and/or migration path on which fish stocks, either within the wetland or elsewhere, depend | Gunbower Creek is an important route for fishes migrating between the river channel and the floodplain. The River Red Gum forests provide habitat via coarse woody debris as well as shade |

Table S3. Status under the Environmental Protection and Biodiversity Conservation Act (EPBC Act) and habitat requirements of threatened species at Macquarie Marshes and Gunbower Forest

| Species | Wetlands | Status under EPBC Act | Habitat Description |
| --- | --- | --- | --- |
| Murray Cod *Maccullochella peelii peelii* | Macquarie Marshes, Gunbower Forest | Vulnerable | Considered a benthic main channel specialist. Found in wide range of habitats: deep, turbid, slow-flowing lowland rivers o fast-flowing, clear streams. Tends not to spawn on inundated floodplains, though may occasionally frequent them (Koehn *et al*. 2020) |
| Silver Perch *Bidyanus bidyanus* | Macquarie Marshes, Gunbower Forest | Critically endangered | From slow-flowing, turbid lowland rivers to faster-flowing reaches; occasionally in floodplain lakes. Spawning occurs in river channels with flowing water (≥0.3 m s^-1^) (Koehn *et al*. 2020) |
| Australasian Bittern *Botaurus poiciloptilus* | Macquarie Marshes | Endangered | Areas of dense vegetation (e.g. rushes and reeds) up to 3.5 m high; water up to 0.3 m deep (DEE 2019a) |
| Australian Painted Snipe *Rostratula australis* | Macquarie Marshes | Endangered | Shallow freshwater wetlands with wet mud and low vegetation of variable height (DEE 2019b) |


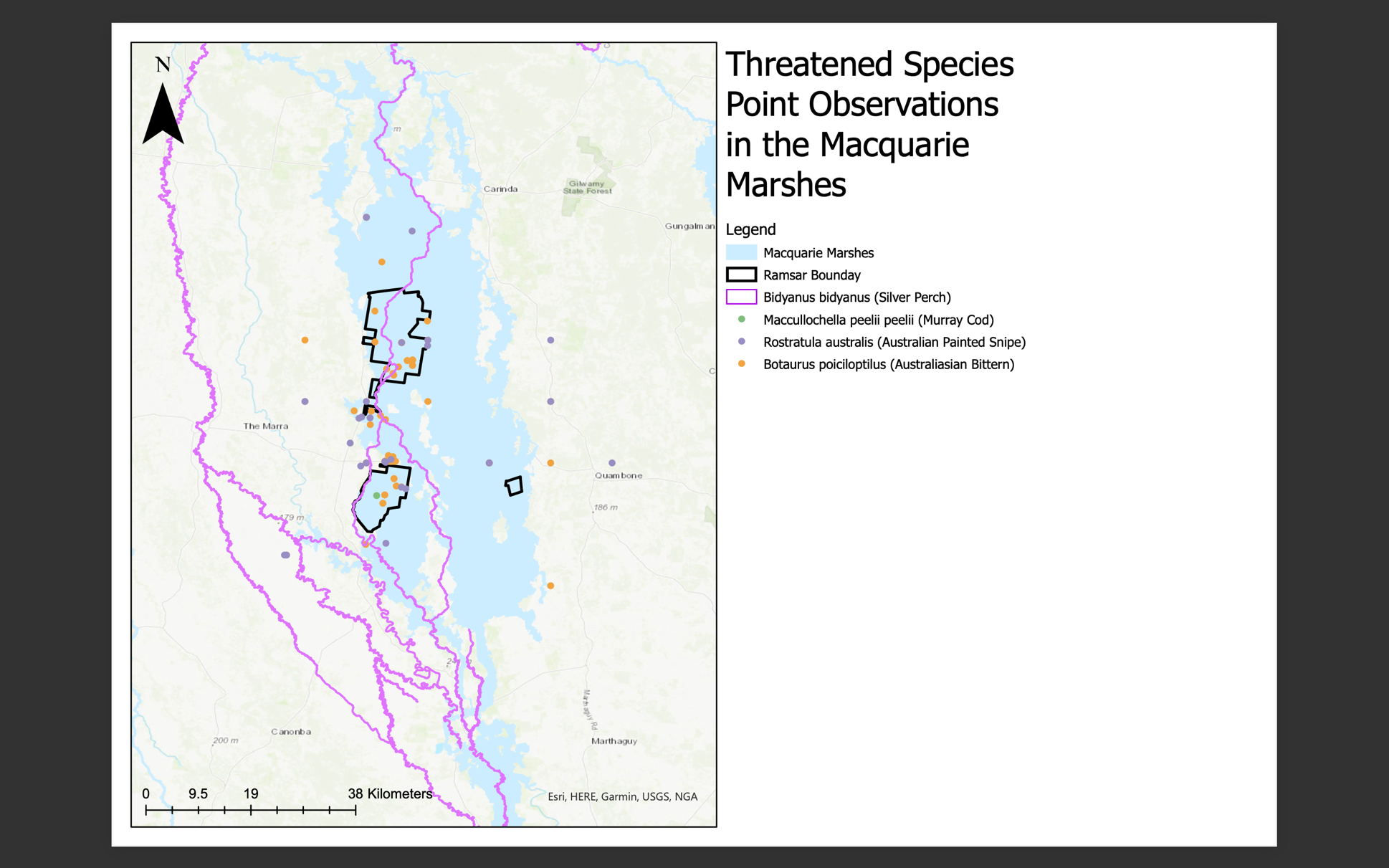
Figure S1. Map showing point source records of threatened species at the Macquarie Marshes.

The Atlas of Living Australia does not include point source data for Silver Perch, so a layer of its known habitat is used to represent occurrence (sources: Atlas of Living Australia <https://www.ala.org.au/>; DPI 2015)

Figure S2. Map showing point observations of threatened species at Gunbower Forest. Note the inclusion of the national park boundary within the site (sources: Atlas of Living Australia <https://www.ala.org.au/>; DPI 2015)

**Section S1. Application of the environmental watering triage framework to case studies**

*Macquarie Marshes*

Figure S5 shows the application of the triage framework to the 2019 environmental watering. The watering and extensive flooding of the Northern Reserve reed beds, is in line with the triage assessment. The reasons the watering meet each consideration in the alternative framework are outlined as follows:

The Northern Reserve reed bed was flooded more often than not between 2016-2019 and the water requirements of the vegetation community were met.

Only 7.5 percent of the Northern Reserve reed bed wetland type ‘aquatic grassland and rushland swamp’ is protected in the Basin (Table 1); well below the 30 percent required to meet Achi targets (CBD, 2009). The Northern Reserve reed beds are within the Northern Nature Reserve Ramsar site boundary, so the watering of this area helps meet the Ramsar listing criteria 1 and 3 for the Macquarie Marshes. Previous environmental watering has supported the North Marsh reedbed (CEWO, 2021c). This statement, along with existing environmental works in the area, indicates the ability to target environmental water to this site.

Finally, through this watering, not only will the ecological watering requirements of two wetland vegetation communities (common reed and water couch) be met but, depending on the water available, native fish could also be supported (CEWO, 2021a). The watering of this area is also important to the Traditional Owners. The North Marsh Nature Reserve is a culturally significant place, and following flooding in 2012, reeds were collected as part of a cultural camp run by Aboriginal women for the sharing of stories and knowledge (Mackay, 2016).


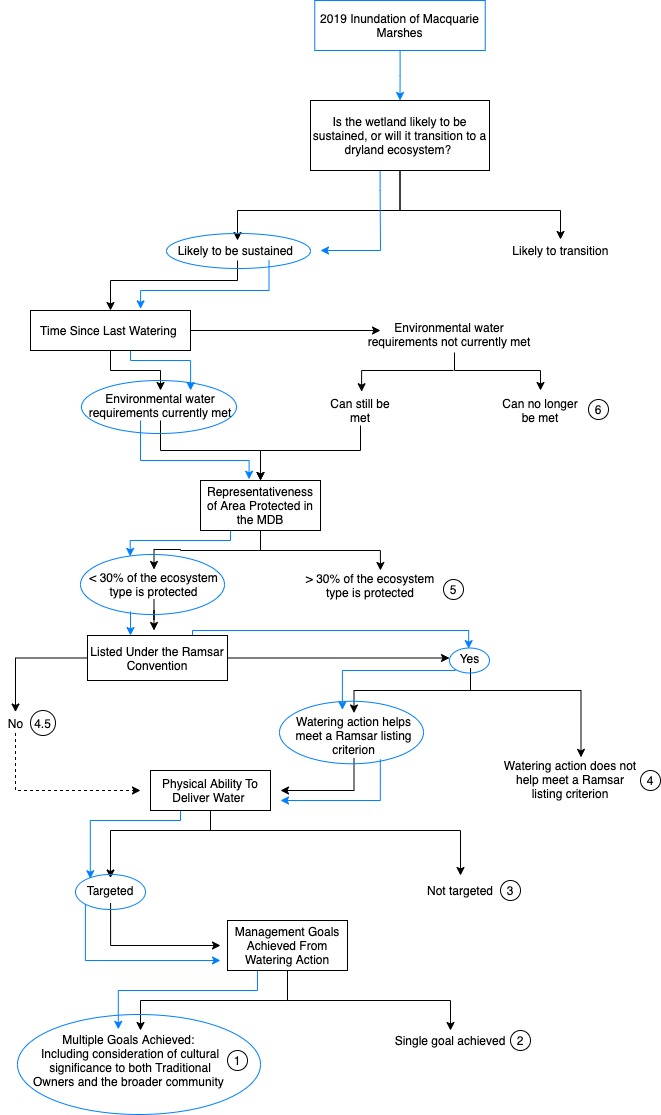


Figure S5. Application of the triage framework to 2018 environmental flows at the Macquarie Marshes

*Gunbower Forest*

An assessment of the 2019 watering indicates that the event was not consistent with my triage framework when limited environmental water is available. The three wetlands in Gunbower Forest inundated in 2019 (Reedy Lagoon, Little Gunbower Wetland and Black Lagoon) consist of river red gum vegetation communities which have had their environmental watering requirements met. As this community has greater than 30 per cent of its distribution within the MDB protected it is not considered a priority for environmental watering under the triage framework.

*
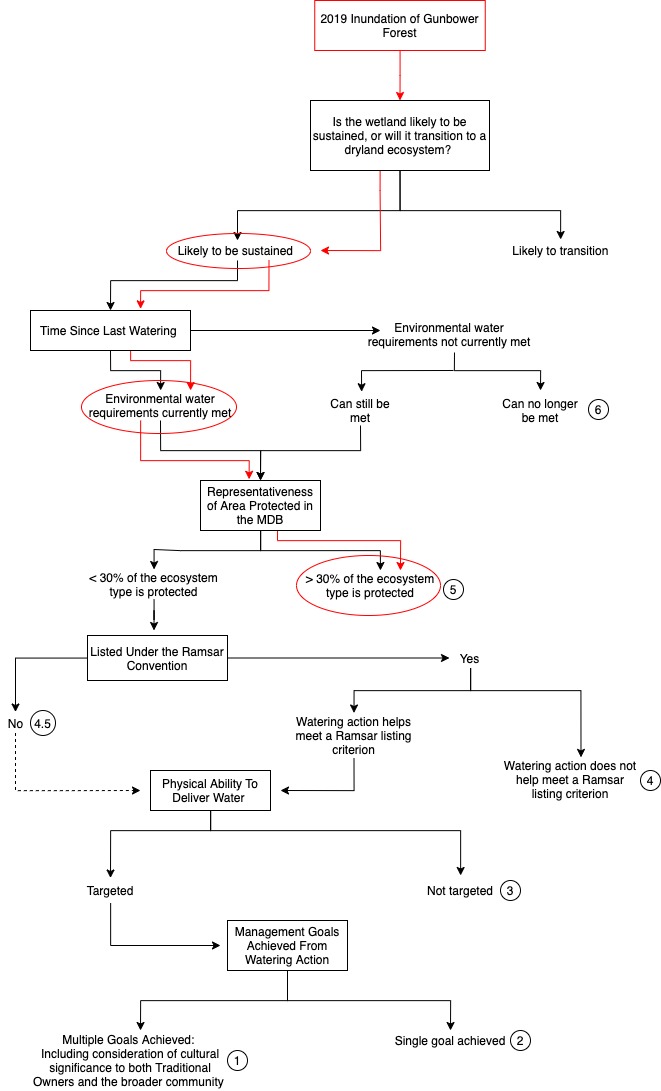
*

Figure S6. Application of the triage framework to 2019 environmental watering at Gunbower Forest

References

DEE (2019a) Draft national recovery plan for the Australasian Bittern (*Botaurus poiciloptilus*). Department of the Environment and Energy, Canberra. Available at: <https://www.awe.gov.au/sites/default/files/env/consultations/9a03b781-7f67-4874-a919-cf53cd1eee60/files/draft-recovery-plan-australasian-bittern.pdf> [accessed 3 April 2022]

DEE (2019b) Draft national recovery plan for the Australian Painted Snipe (*Rostratula australis*). Department of the Environment and Energy, Canberra. Available at: <https://www.awe.gov.au/sites/default/files/env/consultations/5e6b3fbf-ef4d-4d0a-b9c8-c8e29bb11afc/files/draft-recovery-plan-australian-painted-snipe.pdf> [accessed 3 April 2022]

DPI, 2015. Silver Perch indicative distribution in Murray–Darling Basin NSW. Department of Primary Industries, Sydney. Available at: <https://datasets.seed.nsw.gov.au/dataset/silver-perch-indicative-distribution-in-murray-darling-basin-nsw-0> [accessed 5 April 2022]

Koehn, J., Raymond, S., Stuart, I. G., Todd, C., Balcombe, S., Zampatti, B. P., Bamford, H., Ingram, B., Bice, C., Burndred, K., Butler, G. L., Baumgartner, L. J., Clunie, P., Ellis, I., Forbes, J., Hutchison, M., Koster, W., Lintermans, M., Lyon, J., Mallen-Cooper, M., McLellan, M., Pearce, L., Ryall, J. G., Sharpe, C., Stoessel, D., Thiem, J., Tonkin, Z., Townsend, A. & Ye, Q. (2020). A compendium of ecological knowledge for restoration of freshwater fishes in Australia's Murray–Darling Basin. *Marine and Freshwater Research* 71, 1391–1463.
